# High-separation efficiency micro-fabricated multi-capillary gas chromatographic columns for simulants of the nerve agents and blister agents

**DOI:** 10.1186/1556-276X-9-224

**Published:** 2014-05-08

**Authors:** Yi Li, Xiaosong Du, Yang Wang, Huiling Tai, Dong Qiu, Qinghao Lin, Yadong Jiang

**Affiliations:** 1 State g Laboratory of Electronic Thin Films and Integrated Devices, School of Optoelectronic Information, University of Electronic Science and Technology of China (UESTC), Chengdu 610054, People's Republic of China

**Keywords:** Micro-fabricated multi-capillary gas column, Separation efficiency, Flow splitters, High-speed separation, CWA simulants

## Abstract

To achieve both high speed and separation efficiency in the separation of a mixture of nerve and blister agent simulants, a high-aspect-ratio micro-fabricated multi-capillary column (MCC, a 50-cm-long, 450-μm-deep, and 60-μm-wide four-capillary column) was fabricated by the application of the microelectromechanical system (MEMS) techniques. Mixtures of chemical warfare agent (CWA) simulants - dimethyl methylphosphonate (DMMP), triethyl phosphate (TEP), and methyl salicylate - were used as samples. The fabricated MCC allowed for the separation of all the components of the gaseous mixture within 24 s, even when the difference in boiling point was 4°C, as in the case of TEP and methyl salicylate. Furthermore, interfering agents - dichloromethane, ethanol, and toluene - were also included in the subsequent gaseous mixture samples. The boiling point of these six components ranged from 78°C to 219°C. All six components were clearly separated within 70 s. This study is the first to report the clear separation of gas mixtures of components with close boiling points. The column efficiency was experimentally determined to be 12,810 plates/m.

## Background

Since Terry's first report in 1979 [1], micro-fabricated gas chromatography (GC) columns have been developed for over 30 years. The new generation of GC columns has unique characteristics. Silicon is often used as a substrate for column fabrication. These GC columns come in small sizes with high-column efficiency [2] and differ significantly from packed or capillary columns, which are made of steel or silica [3,4]. Thus, micro-fabricated columns are suitable for applications in hand-held GC systems [5]. The structure of the GC column varies when fabricated via microelectromechanical system (MEMS) processes. For instance, since the depth and width of columns can be arbitrarily designed, the column structure can feature different aspect ratios. These flexibilities provide a new direction for research in this field.

Over the past 30 years, techniques for column fabrication have changed significantly. Wet etching was an important technique in early fabrication techniques [6]. In 1998, Sandia National Laboratories reported the application of wet etching process to fabricate single open-tube columns with rectangular channels [7]. However, precise regulation of concentrations and temperatures of etching solution were important factors that influenced structure formation. The chemical wet etching technique has not found widespread use because of its lack of control over the structure. To allow for better control of the column shape, the deep reactive-ion etching (DRIE) technique was developed. This technique prevents lateral etching of the silicon and results in highly anisotropic etch profiles at high etch rates [8]. Etching capabilities can vary from <1 μm to >700 μm in depth in vertical sidewalls [9]. Considering its many advantages, DRIE has become the workhorse of column fabrication.

Since the 9/11 attack, acts of terrorism have become a matter of significant concern to many countries. Chemical warfare agents (CWAs) constitute one class of such lethal weapons for potential use by terrorists. Rapid separation and identification of lethal gas in public space is a great challenge, especially in airports and subways. Previously, researchers have shown that micro-fabricated GC columns can separate the components of a mixture in a complex environment [10,11]. For instance, MEMS-based semi-packed GC columns can separate environmental carcinogens with concentrations at the ppb level [12] with higher separation efficiency than commercial GC columns, and the total length of the GC column is only 2-m long.

To reduce the retention time of analytes, extra-short GC columns can be used. Agah et al*.*[13] designed a high-speed signal open-tube GC column, through which components of the mixture were separated within 10 s. However, the separation efficiency and sample capacity of the fabricated column can be improved further. In 1975, Golay introduced the principle of multi-capillary columns (MCCs). MCCs demonstrated much higher sample capacities when compared with single capillary column [14,15]. MEMS-based multi-capillary GC columns were subsequently designed. The sample capacity of MCC was ten times higher than in the single channel [16]. However, for MCCs with a short length, the separation efficiency needs to be improved further. Our work focuses on improving separation efficiency by designing a column with a high aspect ratio.

In this study, MEMS techniques were applied in the fabrication of an MCC. Using the DRIE process, a 50-cm-long, 450-μm-deep, and 60-μm-wide four-capillary column was fabricated. The static coating method was used for coating the column with the stationary phase - dimethyl (94%) + vinyl (1%) + phenyl (5%) polysiloxanes (SE-54). Mixtures of DMMP, TEP, and methyl salicylate (representing CWAs) were used as samples to evaluate the efficiency of the column. Dichloromethane, ethanol, and toluene were added as interference components to the analytes to produce new sample mixtures.

## Methods

### Materials and reagents

A solution of SE-54 (5% phenyl, 1% vinyl, 94% dimethyl polysiloxane) was purchased from Sigma-Aldrich (St. Louis, MO, USA) for use as the stationary phase. The internal unions were purchased from VICI (Valco Instruments Co., Schenkon, Switzerland), and the fused silica tubing was purchased from SGE (SGE Analytical Science, Ringwood, VT, Australia). All analytes were purchased from J&K Scientific Ltd. (Beijing, China). Samples (mixture of gases) were generated by a MF-3C dynamic vapour generator, where the analyte-solvent mixtures were injected into a vaporising chamber. Two digital mass flow controllers in the vapour generator regulated the concentration of the sample.

### MEMS fabrication

**Figure 1:**
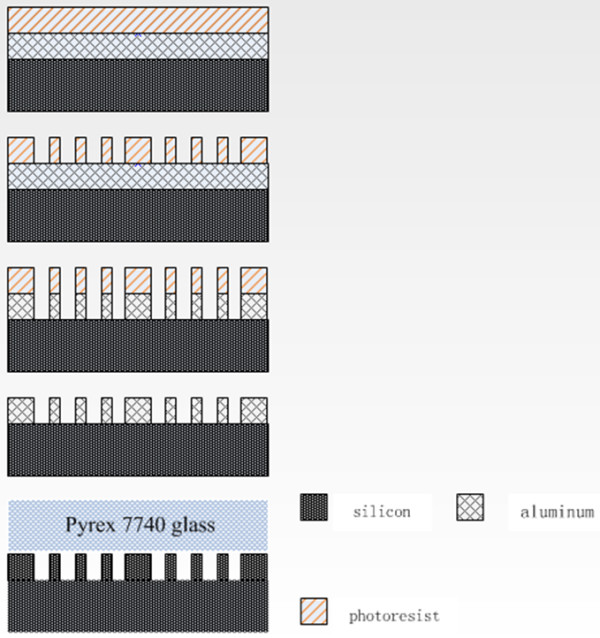
Multi-capillarycolumn fabrication process.

**Figure 2:**
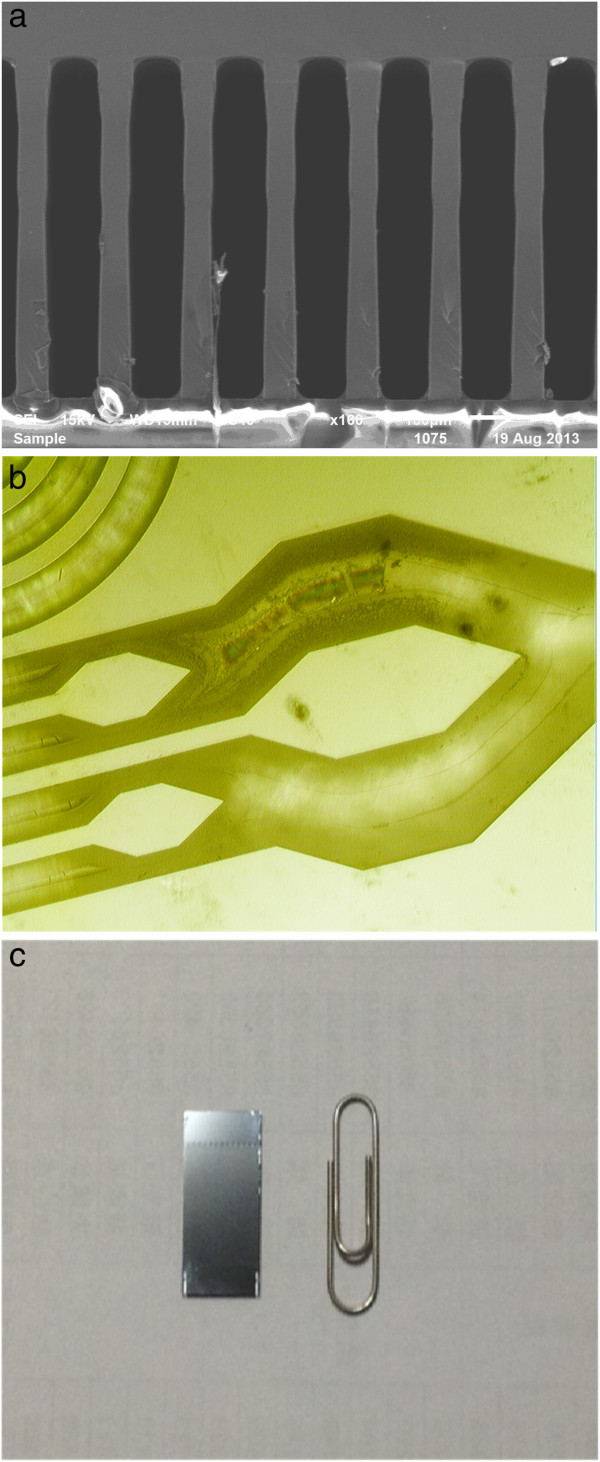
**Structural features of MCC. (a)** SEM image of the crosssection of MCC, **(b)** the flow splitters at the inlet of MCC, **(c)** size of the MCC; the length and width of the chip are 2.5 cm × 1.2 cm.

The DRIE technique was applied to create an MCC with 7.5:1 aspect ratio (length = 50 cm, depth = 450 μm, and width = 60 μm). The steps involved in MCC fabrication is shown in Figure 1. The aluminium film was deposited on type <100 > silicon wafer by electronbeam evaporation. The thickness of the aluminium film was approximately 3 μm. The photoresist was then coated on the wafer (4-μm-thick layer) and patterned as an etch mask for aluminium. The etchant was used to wash the parts of unprotected aluminium film, thereby exposing the silicon surface underneath. The DRIE etching process was then performed by introducing the two gases (sulphur hexafluoride, SF_6_, and octafluorocyclobutane, C_4_F_8_) alternately into the chamber. SF_6_etched the silicon while C_4_F_8_ formed a passive layer [17]. The channels formed vertical sidewalls via this technique. Figure 2a shows the MCC structure. In addition, to ensure that the carrier gas was partitioned equally through the capillaries of an MCC, i.e. the carrier gas must have the same velocity as it travels through each capillary, flow splitters were created at the inlet and outlet of the MCC which is shown in Figure 2b. Finally, the aluminium mask was stripped off and the column was sealed by bonding Pyrex 7740 glass to the silicon wafer as shown in Figure 2c.

### Coating procedure

#### Deactivation

The MCC was deactivated with octamethylcyclotetrasiloxane (D4) before coating with the stationary phase. Since silanol (Si-OH) groups can attract moisture on the surface through hydrogen bonding and influence column performance, D4 was used to remove Si-OH groups and inactivate the surface of the column [18,19]. D4 was injected into the MCC and both ends of the column were sealed. To ensure complete deactivation, the column was placed in an oven at 400°C for 90 min. After deactivation, the GC column was washed with methylene chloride (1 mL) while using N_2_ as carried gas at 220°C for 60 min to remove all residues.

#### Coating

**Figure 3:**
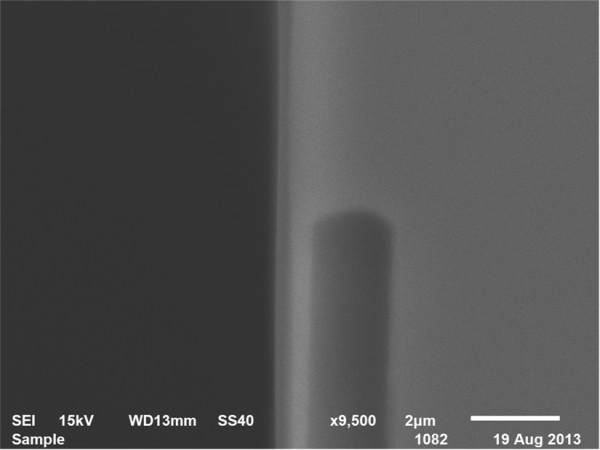
SEM images of the middle of the column wall of MCC after coating.

SE-54 was used as the stationary phase. A solution of the stationary phase material consisted of 5% polar phase (0.16 g) in 1:1 (*v*/*v*) mixture of n-pentane and dichloromethane (2.0 mL). The vial containing this solution was sonicated for 30 min. One end of the fused silica connecting line was connected to a vacuum pump and the other end was sealed by wax. The MCC was maintained at 38°C in a water bath and the solution of the stationary phase pumped through it for 2 h (pressure of the columns = 12 KPa). Subsequently, methyl groups present in the column were treated with ozone to form free radicals and readily cross-link to form a more stable, higher-molecular weight gum phase [15,20]. Ozone, produced by an ozone generator, was passed through the column for 25 min. Subsequently, the two open ends of the fused silica were sealed and the column was kept at room temperature for 20 min. The MCC was washed by N_2_ for 3 h. After cross-linking, the temperature of the column was increased at a rate of 5°C/min until it reached 180°C; the column was kept at 180°C for 4 h. Figure 3 shows an image of the column after coating.

## Results and discussion

### Flow splitters

**Figure 4:**
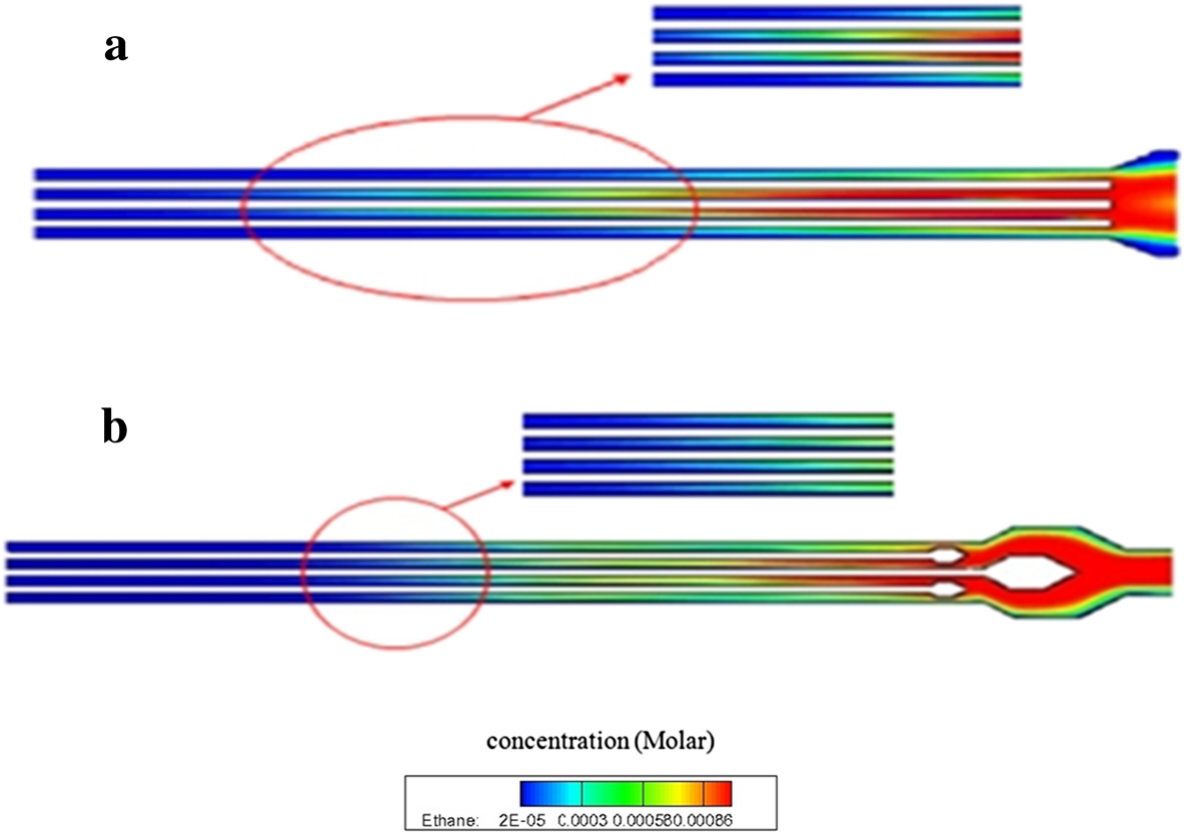
Distribution of ethane flow through inlet of multi-capillary column: (a) multi-capillary column with and (b) without flow splitters.

To ensure that the sample gas is partitioned equally into each channel of the MCC, flow splitters were designed (Figure 2b). The initial large splitter divides the sample gas equally into two parts; the two subsequent splitters further divide the sample into each of the four channels. The effectiveness of the flow splitter, as simulated by ANSYS FLUENT, is evident from Figure 4a,b. In the simulation, ethane, used as a sample gas, is injected into the inlet with a velocity of 17 cm/s. Figure 4b shows the MCC without flow splitters; the sample flows slower in the top and bottom channels than in the two middle channels. After the addition of the splitters, the sample gas flows equally in all the four channels (Figure 4a).

### Film thickness of the stationary phase

Two main methods are used in coating procedures, i.e., static and dynamic. Dynamic coating is performed by pushing the solution of the stationary phase material through the column with a carrier gas, where in the film thickness, depends on the velocity and concentration of the stationary phase. In static coating, the column is filled with the stationary phase solution and slow evaporation of the solvent is allowed to take place, thus leaving the stationary phase behind. Static coating allows for tailoring of the film thickness because the method does not involve flow velocity. Film thickness resulting from static coating can be calculated using Equation 1, which divides the total coating mass dissolved in the solution by the total column internal surface [14]. The film thickness *d*_*f*_ can be expressed as

(1) $$d_{f}\frac{C_{\mathrm{cs}}}{2\rho_{\mathrm{stationaryphase}}}\frac{1}{\left(\frac{1}{h}+\frac{1}{w}\right)}$$

where *C*_cs_ is the coating solution concentration; *ρ*_statonary phase_ is the stationary phase density; and *w* and *h* are the channel width and height, respectively. In this experiment, the film thickness was controlled to approximately 1 μm using static coating. Figure 3 shows the film thickness in the middle of the channel.

### Column efficiency

#### Theoretical determination of column efficiency

The separation efficiency of single capillary chromatographic columns can be defined by the height equivalent to a theoretical plate (HETP), expressed in Equation 2 [19].

(2) $$\begin{array}{l} \mathrm{HETP}=\frac{2D_{g}}{u}f_{1}f_{2}+\frac{1+9k+25.5k^{2}}{105{\left(k+1\right)}^{2}}\frac{w^{2}}{D_{g}}\frac{f_{1}}{f_{2}}u\\ \;+\frac{2}{3}\frac{k}{{\left(k+1\right)}^{2}}\frac{{\left(w+h\right)}^{2}d_{f}^{2}}{D_{s}h^{2}}u \end{array}$$

where *d*_*f*_ is the stationary phase thickness; *w* and *h* are the channel width and height, respectively; *D*_*g*_ and *D*_*s*_ are the binary diffusion coefficients in the mobile and stationary phases, respectively; and *f*_1_ (varies between 1 and 1.125) and *f*_2_ (varies between 0 and 1) are the Gidding-Golay and Martin-James gas compression coefficients, respectively.

For MCCs, the HETP is determined by the performance of its single capillaries, stationary phase properties, and structural features. The HETP for MCC can be expressed as Equation 3 [15].

(3) $$\mathrm{HETP}=\frac{u_{0}^{2}}{L{\left(1+k_{0}\right)}^{2}}\sigma_{0}^{2}+L\;{\left(\frac{\sigma_{h}}{h_{0}}\right)}^{2}+L\;{\left(\frac{\sigma_{w}}{w_{0}}\right)}^{2}$$

where $\sigma_{0}^{2}$ is the peak variance; u_0_ is the average linear gas velocity; $\frac{\sigma_{h}}{h_{0}}$ and $\frac{\sigma_{w}}{w_{0}}$ are the cross-sectional height *σ*_*h*_ and width *σ*_*w*_ variances normalised by the average height *h*_0_ and average width *w*_0_, respectively; and *k*_0_ is the retention factor in a capillary with some cross-sectional area. In this equation, the first term refers to the HETP of a capillary whose dimensions are the average of the dimensions of all capillaries in the bundle [9]. This value is directly expressed by Equation 2. The second and third terms account for the band broadening caused by non-uniformity in the channels. In this experiment, since each of the four channels has a width of 60 μm and a depth of 450 μm, *σ*_*h*_ and *σ*_*w*_ are equal to 0. Thus, the second and third terms are cancelled and the HETP of an MCC is equal to its single capillary; the sample capacity is simply multiplied by the number of capillaries in the bundle [15].

**Figure 5:**
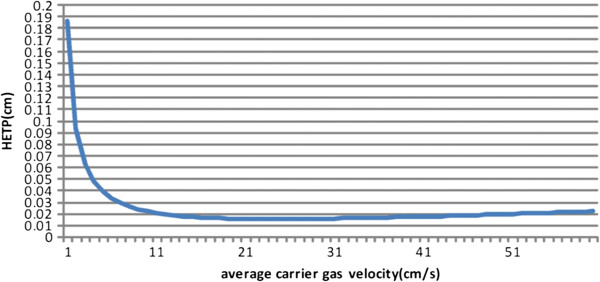
**Height equivalent to theoretical plate (*H*) versus average carrier gas velocity for the four-capillary micro MCC.** Length = 50 cm, depth = 450 μm, and width = 60 μm.

Therefore, Equation 2 can be used to express the HETP of the MCC. Figure 5 shows calculated HETPs versus average carrier gas velocity from Equation 2. *D*_*g*_ and *D*_*s*_ values are chosen as 0.093 cm^2^/s and 6.4 × 10^−6^ cm^2^/s, respectively, and the *k* value is 3. The width, *w*, and height, *h*, of the channel are 60 and 450 μm, respectively. With increasing average carrier gas velocity, the curve drops dramatically and subsequently flattens (Figure 3). This finding indicates that at higher velocities, the column efficiency is not affected significantly with any further increase in the average flowrate of carrier gas. In practical applications, MCCs exhibit stability with variable velocity. With the average carrier gas velocity of 24 cm/s, the HETP of the column is only 0.0151 cm. The column achieves maximum column efficiency at this point.

#### Experimental determination of column efficiency

**Figure 6:**
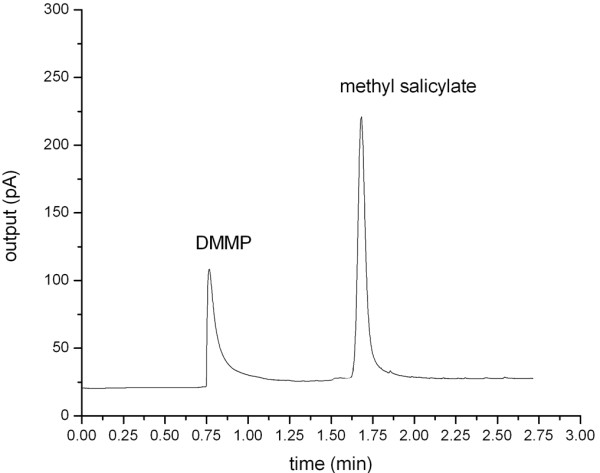
**Separation of two component mixtures: DMMP and methyl salicylate.** Column temperature = 130°C.

In our study, the internal unions and fused silica tubing serve as connectors to mount the MCC on an Agilent GC 6890 system. Heliumis used as a carrier gas. A1:1 (*v*/*v*) mixture of DMMP and methyl salicylate is used as a sample. The inlet temperature is set to 250°C, and the sample volume is 0.1 μL with a split ratio of 200:1. The carrier gas velocity is set to 20 cm/s. The initial column temperature is set at 80°C and programmed to increase at rate of 30°C/min till it reaches 200°C. The observed retention times of DMMP and methyl salicylate are 0.766 and 1.682 min, respectively (Figure 6). The theoretical number of plates can be calculated based on the retention times (*t*_R_) of the peaks [15].

(4) $$n=5.54\;{\left(\frac{t_{R}}{w_{\frac{1}{2}}}\right)}^{2}$$

where $w_{\frac{1}{2}}$ is the width of the peak at half height. The number of plates for methyl salicylate is 6,410 plates. With a 1-m length, the theoretical number of plates is 12,810 plates/m. The main advantage of short-length GC columns is its ability to separate components in a short period of time. Using Equation 4, the shorter retention time of peak, the lower plate number is worked out. Meanwhile, the resolution also deceases when components are eluted quickly from the column. In our design, we optimise MEMS-based MCC separation conditions by striking a balance between the time required for separation, and a rational resolution and plate number.

### Chromatographic separation of mixture components

**Figure 7:**
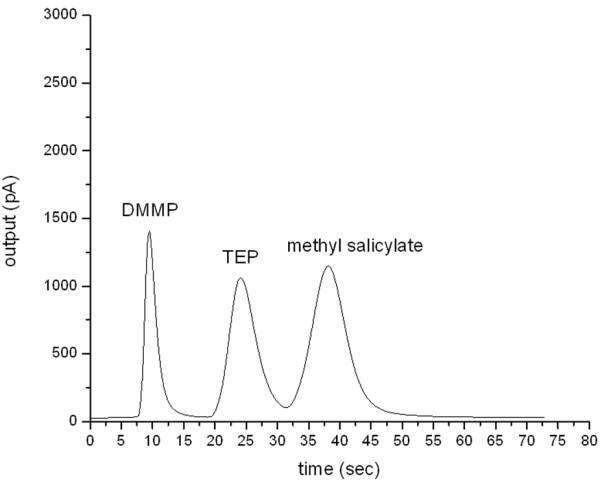
**Separation of the mixture of CWA simulants: DMMP, TEP, and methyl salicylate.** The carrier gas velocity is 18 cm/s.The initial temperature of gas chromatography column is set at 140°C. The temperature of the column was programmed to rise at the rate of 100°C/min till 200°C. The samples were mixtures of CWA simulants with a concentration of 100 ppm each.

Here, because of restrictions associated with the use of CWAs, stimulants are used to test the separation efficiency of the MCC. DMMP and TEP are used as nerve agent simulants, while methyl salicylate serves as a blister agent simulant. The boiling points of TEP and methyl are 215°C and 219°C, respectively, showing a boiling point difference of only 4°C. The vaporised simulants at a concentration of 100 ppm are stored inside an air bag. The carrier gas velocity is set to18 cm/s. About 1 mL of the gas mixture is injected into the Agilent GC 6890 system (Santa Clara, CA, USA) with a 200:1 split ratio. The initial temperature of the GC column is set to 140°C, and the column temperature is programmed to increase at a rate of 100°C/min until it reaches 200°C. Under these conditions, all the gas components are separated within 24 s (Figure 7). The resolutions of the two adjacent peaks are 2.10 and 1.30. Therefore, MCC achieves both high speed and high separation efficiency.

**Figure 8:**
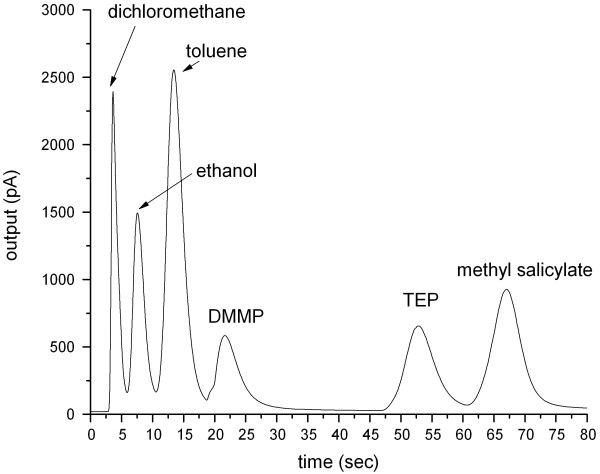
**Separation of six components of a mixture: dichloromethane, ethanol, toluene, DMMP, TEP, and methyl salicylate.** The velocity of the carrier gas is 18 cm/s and the column temperature is 110°C.

In another experiment, interfering components (i.e., dichloromethane, ethanol, and toluene) are also mixed with the simulants to produce a new gas mixture. The boiling points of the six components range from 78°C to 219°C. The concentration for each sample is maintained at 100 ppm, and the column is kept at a constant temperature of 110°C. About 1 mL of the mixture gas is injected into the column at a split ratio of 200:1. The carrier gas velocity is maintained at 18 cm/s. All components are separated within 70 s (Figure 8). The plate numbers of all components are low (Table 1). These results are caused by the low distribution constant of each component in short column length. However, the resolution of each peak is greater than 1.4, which is close to that required for baseline separation (1.5). These results indicate that the MCC possesses a high separation efficiency and can separate components with a wide range of boiling points within a short period of time. Thus, the low plate number of components can be accepted rationally.

**Table 1:** Separation of six components in MCC

**Sample**	**Retention time (min)**	**Number of plates/m**	**Resolution**
Dichloromethane	0.064	116	
Ethanol	0.127	154	1.43
Toluene	0.224	236	1.45
DMMP	0.362	362	1.48
TEP	0.88	1,166	4.09
Methyl salicylate	1.117	1,952	1.64

## Conclusions

In this work, the MEMS technique was used to fabricate a MCC column which was 50-cm long. By applying the DRIE technique, a 60-μm-wide and 450-μm-deep MCC was fabricated; these dimensions resulted in an aspect ratio of 7.5:1. The resulting MCC achieved both high speed and high separation efficiency in separating nerve and blister agent simulants. This study is the first to report MCC etching at such high depths.

Flow splitters were installed at the inlet and outlet of the MCC. By simulating the flow of carrier gas through the column, the gas flow was shown to be equally divided between the capillaries of the MCC. To evaluate the effects of interfering components, we mixed three commonly used chemicals with the simulants. The boiling points of the six components ranged from 78°C to 219°C. This study is the first to report a successful separation of gas mixtures containing components with close boiling points.

This short length of the MCC ensured that components of the mixture were rapidly separated, i.e. within 70 s. The number of plates was determined to be 12,810 plates/m. The results indicate that the proposed MCC will find applications as a new generation of GC columns. The present study also features several limitations. First, fabrication of the MCC entails high costs. Furthermore, a smaller GC system requires miniaturisation of its component devices. Production of MCCs in a batch-to-batch manner may help reduce costs for commercialisation.

## Competing interests

The authors declare that they have no competing interests.

## Authors' contributions

YL and XSD conceived and designed the experiments and wrote the manuscript. YL, YW and HLT performed the experiments. YL, XSD, and YDJ analyzed the data. YL, DQ and QHL contributed reagents/materials/analysis tools. All authors read and approved the final manuscript.
